# Fluorescence Spectroscopy Shows Porphyrins Produced by Cultured Oral Bacteria Differ Depending on Composition of Growth Media

**DOI:** 10.1159/000528731

**Published:** 2022-12-16

**Authors:** Áine M. Lennon, Leonora Brune, Simone Techert, Wolfgang Buchalla

**Affiliations:** ^a^Department of Conservative Dentistry and Periodontology, University Hospital Regensburg, Regensburg, Germany; ^b^Department Oral and Maxillofacial Surgery, University Hospital Regensburg, Regensburg, Germany; ^c^Private practice Soest, Soest, Germany; ^d^Deutsches Elektronen-Synchrotron DESY, Hamburg, Germany; ^e^Institute of X-ray Physics, Göttingen University, Göttingen, Germany

**Keywords:** Fluorescence spectroscopy, Porphyrins, Caries, Periodontitis, Bacteria, Chlorophyll, Haem

## Abstract

Red fluorophores synthesized by oral bacteria are important for fluorescence-based diagnosis and treatment because they are used as markers for bacterially infected tissue, mature plaque, or calculus. A range of porphyrins have been identified as the source of this fluorescence in carious tissue. It is not clear which of these porphyrins are produced by individual oral bacteria or whether this ability depends on other factors. This study examined and compared the fluorescence spectra produced by selected cultured oral bacteria when grown on agars containing different nutrients with spectra for protoporphyrin IX, Zn-protoporphyrin IX, haematoporphyrin, and haematin. *Actinomyces israelii* (Deutsche Sammlung von Mikroorganismen [DSM], 43320)*, Actinomyces naeslundii* (DSM 43013)*, Fusobacterium nucleatum* (DSM, 20482), *Lactobacillus casei* (DSM, 20011), *Prevotella intermedia* (DSM, 20706), *Streptococcus mutans* (DSM, 20523), *Streptococcus oralis* (DSM, 20627), *Streptococcus salivarius* (DSM, 20560) and *Streptococcus sobrinus* (DSM, 20742) were rehydrated and grown anaerobically on caso, caso blood (containing 5% sheep blood), and caso chlorophyll (containing 5% spinach extract) agar for 3 days at 37°C in the dark. Colonies were harvested, transferred to ethanol, and centrifuged. Fluorescence emission spectra were recorded from the supernatant at 405 nm excitation (Fluorolog 3–22, Jobin Yvon-Spex ISA, Edison, NJ, USA). All *Streptococci*, *L. casei*, and *F. nucleatum* produced red fluorescence when grown on caso and caso chlorophyll agar but not on caso blood agar. *A. naeslundii* and *P. intermedia* emitted intense red fluorescence when grown on caso or caso blood agar but not on caso chlorophyll agar. Fluorescence emission spectra of *A. naeslundii* and *P. intermedia* grown on caso blood agar correlated exactly with both fluorescence peaks for protoporphyrin-IX at 632 and 701 nm. Most peaks observed could be correlated with at least one of the emission peaks of protoporphyrin IX, Zn-protoporphyrin IX, or haematoporphyrin. Oral bacteria emitted red fluorescence matching known porphyrins, but this depended on nutrients available in the agar.

## Introduction

A number of dental diagnostic tools, for example, fluorescence-aided caries excavation (FACE), QLF^TM^, DIAGNOdent^TM^, SOPROlife®, VistaCam®, SiroInspect^TM^, and Fluoresce HD^TM^, are based on detection of red fluorescence produced by bacteria in the mouth [Lennon et al., 2002; Coulthwaite et al., 2006; Kim, and Kim, 2017; Michou et al., 2020]. Their applications range from detection of various forms and stages of caries, plaque, and calculus, as well as infected pulp tissue, to identification of cracked teeth [Sainsbury et al., 2009; Ku et al., 2022]. They differ in their working principles and the wavelengths used for excitation or detection. Some produce a numerical score, while others produce an enhanced camera image or allow the operator to view the fluorescence directly through a filter. In addition to diagnostics, antimicrobial phototherapies are also possible when endogenously produced fluorophores serve as photosensitizers to allow photodynamic inactivation of bacteria [Cieplik et al., 2014].

These diagnostic and therapeutic methods assume that red fluorescence will be produced by active oral bacteria found in carious tissue, mature plaque, calculus, or infected pulpal tissue. However, there are conflicting reports as to which oral species indeed produce red fluorescence and which do not [Koenig and Schneckenburger, 1994; van der Veen et al., 2006]. Although much is now known about the complexity and taxonomic diversity of the oral microbiome (who they are), their metabolic activities (what they are doing) remain poorly understood [Takahashi, 2015]. It has been shown that oral microorganisms produce not only red but also green fluorescence with green fluorescence found predominantly in early colonisers and red fluorescence in species characteristic of deeper lesions and periodontal pockets [Lennon et al., 2006]. A shift toward a redder and less green fluorescent biofilm indicates a dysbiotic change in composition of the biofilm which can be predictive of oral disease [Volgenant et al., 2016]. One reason for this shift may be the high concentration of iron available in the crevicular fluid produced during gingivitis and periodontitis [Mukherjee, 1985].

Red autofluorescence in caries has long been attributed to porphyrins produced by oral microorganisms. More recently, the presence of porphyrins has been confirmed using high-performance liquid chromatography (HPLC), both in carious dentin and for individual bacterial species grown in culture [Buchalla et al., 2008; Fyrestam et al., 2015]. Porphyrins and protoporphyrins are metabolic intermediates produced by microorganisms in the biosynthesis of haem (ferriprotoporphyrin IX), a tetrapyrrole structure coordinating iron at its centre. Haem is essential for bacterial pathogens not only as a nutritional source of iron but also because of its electron transport capability for a variety of cellular functions, so these microorganisms need to synthesize haem or acquire it from the host [Choby and Skaar, 2016]. Gram-positive and gram-negative bacteria not only use different pathways to synthesise haem, they also use different methods to transport haem into and out of the cell [Mayfield et al., 2011]. In the last decade, new research has identified a third non-canonical pathway used by gram positive bacteria using a coproporphyrin rather than protoporphyrin intermediate for haem biosynthesis [Dailey et al., 2017]. Differences in microbial haem synthesis and transport systems are beginning to be explored as targets for antimicrobials [Shisaka et al., 2019].

At present, little is known about how specific porphyrin synthetic pathways in oral microbes might be affected by the substrates available in culture media or in the mouth. Some oral bacteria may not produce porphyrins in vitro when a required substrate is missing. For other species, synergism with another microorganism may be crucial [van der Veen et al., 2006]. A better understanding of porphyrin metabolism, first in individual oral microbes and then in biofilms, is needed, not only so that species studied in the laboratory have the optimal substrates needed to fluoresce but also to further the development of new diagnostic and selective antimicrobial strategies targeting haem synthesis and transport systems in the future.

One group has shown that different culture media effect fluorescence of individual oral microbes [Volgenant et al., 2013]. Although the porphyrin content of *Aggregatibacter actinomycetemcomitans* and *Porphyromonas gingivalis* has been determined using liquid chromatography, it is not known which specific porphyrins are produced by the wider range of oral bacteria and which nutrients they need to do so [Fyrestam et al., 2015]. The aim of this study was firstly to examine the fluorescence spectra produced by commonly found potentially pathogenic oral bacteria grown on culture media containing different porphyrin precursors and secondly to determine if spectral peaks for specific porphyrins were identifiable depending on which culture medium was used.

## Materials and Methods

### Bacterial Recultivation

The following species were obtained as dried cultures from the Deutsche Sammlung von Mikroorganismen und Zellkulturen (Braunschweig, Germany):

*Actinomyces israelii* (DSM, 43320).

*Actinomyces naeslundii* (DSM, 43013).

*Fusobacterium nucleatum* (DSM, 20482).

*Lactobacillus casei* (DSM, 20011).

*Prevotella intermedia* (DSM, 20706).

*Streptococcus mutans* (DSM, 20523).

*Streptococcus oralis* (DSM, 20627).

*Streptococcus salivarius* (DSM, 20560).

*Streptococcus sobrinus* (DSM, 20742).

All species were rehydrated in 0.1% thioglycolate broth (108191, Merck Millipore, Darmstadt, Germany) and incubated at 36°C. An aliquot of each species was plated in triplicate onto each of three different agar plates (9 plates in total per species), placed immediately in anaerobic jars (Anaerocult, Merk Millipore, Darmstadt, Germany), covered with aluminium foil to exclude light, and incubated for 3 days at 37°C. Anaerobic conditions in the jars were ensured using anaerobic packs: GENbox anaer generator ref. 96124, bioMérieux, Mercy-l'Etoile, France. The agars used were caso standard agar poured using 40 g caso agar base (105458, Merck Millipore, Darmstadt, Germany) to 1 L distilled water for 1 L agar. Caso blood agar containing 5% defibrinated sheep blood (Institute for Medical Microbiology, University of Göttingen, Germany) and caso chlorophyll agar containing 5% spinach extract. The spinach extract was prepared by liquidizing and filtering fresh spinach. The filtrate was centrifuged for 20 min at 3,500 r/min. 50 mL of sterile filtered supernatant and 950 mL of sterile distilled water were used to produce 1 L caso chlorophyll agar. Optical density at 600 nm for the spinach extract was 0.49 (Ultrospec 3,300 pro, Amersham Biosciences, Freiburg, Germany).

### Preparation of Bacterial Samples for Fluorescence Spectroscopy

The colonies were harvested using sterile plastic inoculating loops and placed in Eppendorf tubes (SafeSeal microtubes 2 mL, Sarstedt AG & Co., Nürnberg, Germany) with 1 mL absolute ethanol. The samples were homogenized ultrasonically (Branson Sonifer 250, Heinemann Ultraschall, Schwäbisch-Gmünd, Germany) and then centrifuged at 400 U/min for 5 min. The supernatant was pipetted into a second Eppendorf tube before being transferred to a cuvette for use in the fluorescence spectrophotometer. Samples for the solvent and agar plates on which no bacteria were grown were prepared in the same way as controls.

### Preparation of Porphyrin Samples for Fluorescence Spectroscopy

The following porphyrin samples were also prepared for comparison with the bacterial samples in the fluorescence spectrometer: Zn-II-protoporphyrin IX, porcine haematin (Fe-(III)-protoporphyrin-IX-OH), haematoporphyrin, and protoporphyrin IX (Sigma Aldrich Chemie GmbH, Steinheim, Germany). Ca. 80 μg of each porphyrin compound was dissolved in 1 mL 99.9% ethanol in an Eppendorf tube and then ultrasonicated for 3 min.

### Fluorescence Spectroscopy

Emission spectra were recorded using a modular fluorescence spectrophotometer (Fluorolog 3–22, JOBIN YVON-SPEX Instruments SA, Edison, New Jersey, USA) with a 450 W xenon discharge lamp and a double monochromator to produce a quasi-monochromatic excitation radiation. The excitation radiation was directed at a quartz cuvette (Hellma, quartz glass cuvette SUPRASIL, 114F-QS, pathlength 10 mm, 1 mL). The emission path was 90° to the excitation path, spectrally split by a second double monochromator, and fed into a photomultiplier. The photomultiplier signal was recorded and transferred to a Microsoft Excel table. The parameters for the spectrophotometer were controlled using the DataMax 2.20 software package (Instruments SA, Inc., USA). The spectral data were processed and plotted graphically using Origin version 7 (Microcoal Origin, Microcoal Software, Inc., Northampton, MA, USA) and Microsoft Excel (Microsoft, Redmond, WA, USA).

The emission generated by excitation at 405 nm was scanned from 430 to 800 nm at 0.5 nm/s and recorded with a resolution of 1 nm. Slit width was set between 1 and 6 nm for excitation and emission. The spectra were corrected for excitation intensity, detector sensitivity, and spectral curve of the emission monochromator according to the derived protocols for biochemical fluorescence and emission analysis [Ramos and Techert, 2003, 2005].

## Results

### Emission Spectra of Ethanol, Growth Media, and Selected Porphyrins

Ethanol did not fluoresce but exhibited Raman scattering at 459–460 nm. All three agars used exhibited Raman scattering at 460 nm and green fluorescence with a peak between 504 nm and 509 nm. None of the growth media fluoresced in the red spectral region.

**Fig. 1:**
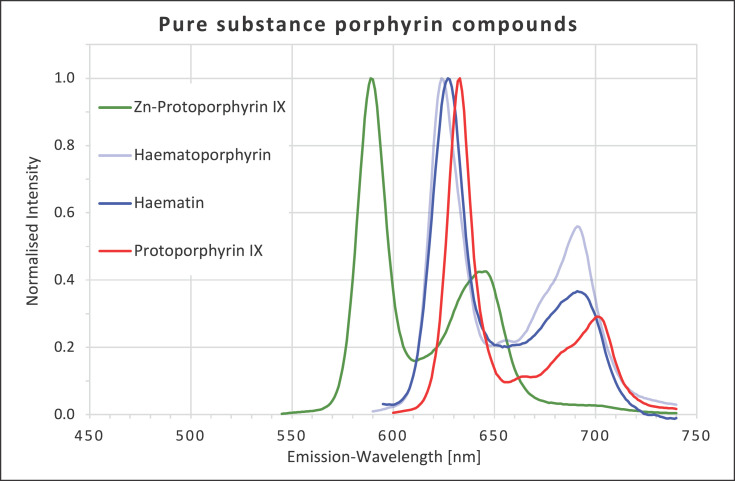
Fluorescence emission spectra for protoporphyrin IX, Zn-protoporphyrin IX, haematoporphyrin, and haematin at 405 nm excitation.

All the porphyrins investigated exhibited strong fluorescence in the red spectral region. Haematin and haematoporphyrin showed very similar first emission peaks at 630 and 624 nm, respectively. The second peak for haematin was at 693 nm and that for haematoporphyrin at 691 nm. Protoporphyrin IX had two clear emission peaks in the same range at 633 and 701 nm. Zn-protoporphyrin IX, on the other hand, exhibited peaks at 589 and 646 nm (Fig. 1).

### Fluorescence Emission from Bacterial Colonies

All bacterial emission spectra showed both a Raman peak for ethanol at 459–460 nm and a broad band of green fluorescence from the growth media between 504 and 509 nm. Fluorescence peaks were observed within three bands, 570–590 nm, 620–635 nm, and 680–700 nm which corresponded with the fluorescence observed for the porphyrins tested.

### P. intermedia

#### Caso Blood Agar

**Fig. 2:**
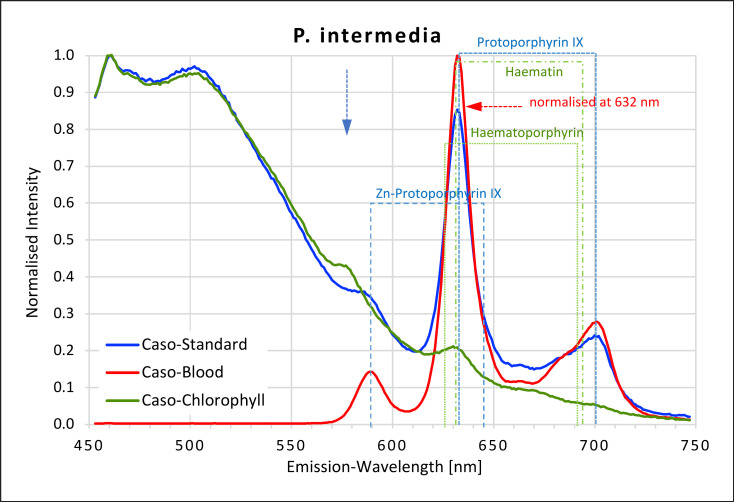
Fluorescence emission spectra for *P. intermedia* at 405 nm excitation on caso, caso blood, and caso chlorophyll agars. The intensity on caso blood agar is normalised at 632 nm. The position of peaks measured for protoporphyrin IX, Zn-protoporphyrin IX, haematoporphyrin, and haematin are shown in dotted lines. Blue arrow at 577 nm.

*P. intermedia* grown on caso blood agar produced the highest intensity red fluorescence recorded in this study (Fig. 2.) Note the intensity on caso blood agar in this figure is normalised at 632 nm = 1 to show the spectrum together with those for the other two agars. The three peaks recorded were at 589–590, 632–633, and 700 nm. The first peak matches the 589-nm peak recorded for Zn-protoporphyrin. The second and smaller peak from Zn-protoporphyrin at 646 nm was not visible but may be masked by the higher intensity peak at 632–633 nm. This peak was an exact match for the first peak found for protoporphyrin IX. The peak at 700 nm is a clear match for the second protoporphyrin IX peak.

#### Caso Standard Agar

When grown on caso standard agar, strong red fluorescence was also found, although the intensity was not as high as when grown on caso blood agar. Again, 3 peaks were found. The second two also matched both peaks for protoporphyrin IX, while the first was a plateau between 575 and 590 nm, corresponding to the first peak for Zn-protoporphyrin.

#### Caso Chlorophyll Agar

In contrast, when grown on caso chlorophyll agar, red fluorescence was inhibited or very weak. Nevertheless, a slight plateau between 570 and 580 nm and a small peak at 631 nm matching the first peaks from haematin and protoporphyrin IX were found.

### Actinomyces

**Fig. 3:**
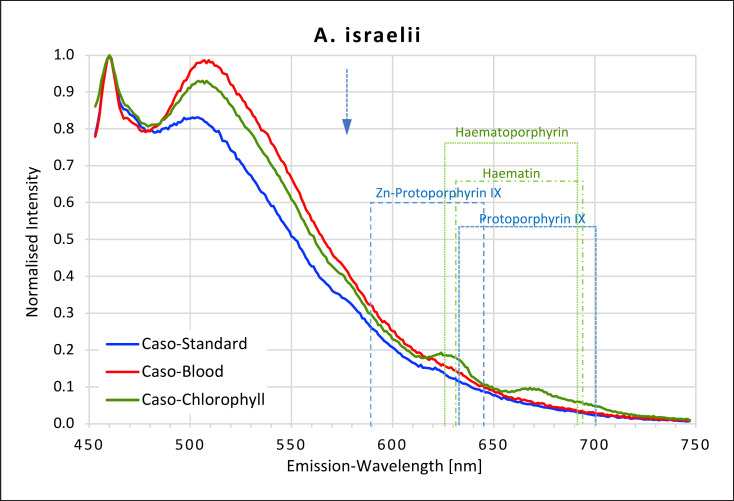
Fluorescence emission spectra for *A. israelii* at 405 nm excitation on caso, caso blood, and caso chlorophyll agars. The position of peaks measured for protoporphyrin IX, Zn-protoporphyrin IX, haematoporphyrin, and haematin are shown in dotted lines. Blue arrow at 577 nm.

**Fig. 4:**
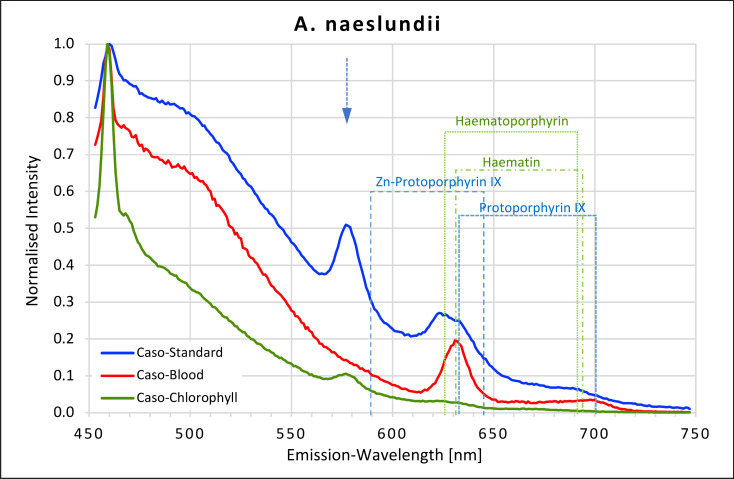
Fluorescence emission spectra for *A. naeslundii* at 405 nm excitation on caso, caso blood, and caso chlorophyll agars. The position of peaks measured for protoporphyrin IX, Zn-protoporphyrin IX, haematoporphyrin, and haematin are shown in dotted lines. Blue arrow at 577 nm.

The two actinomyces species studied showed very different fluorescent behaviour depending on which agar was used (Fig. 3, 4). No fluorescence peaks were recorded for *A. israelii* when grown on either caso agar or on caso blood agar. But when *A. israelii* was grown on caso chlorophyll agar, it fluoresced in the red region, showing a small plateau at 624–630 nm, likely corresponding to the peaks found for haematoporphyrin and haematin and another small plateau at 666 and 676 nm, which did not match any of the porphyrins measured. *A. naeslundii*, on the other hand, fluoresced strongly in the red region on both caso blood and caso standard agars but only very weakly on caso chlorophyll agar. On caso blood agar, a high intensity peak at 631 nm corresponding to haematin and protoporphyrin IX and a second smaller peak at 699–701 nm corresponding to protoporphyrin IX were found.

On caso standard agar, *A. naeslundii* produced three peaks. The first distinctive peak at 577 nm did not match any of the porphyrins tested; a second shoulder between 621 and 632 nm corresponded to those found for haematoporphyrin, haematin, and protoporphyrin IX; and a third small plateau at 689–690 nm matched the second peak recorded for haematoporphyrin. On caso chlorophyll agar, the 577 nm peak was visible and a very slight shoulder at 622–626 nm was present, similar to the first two peaks found on caso standard agar.

### F. Nucleatum

**Fig. 5:**
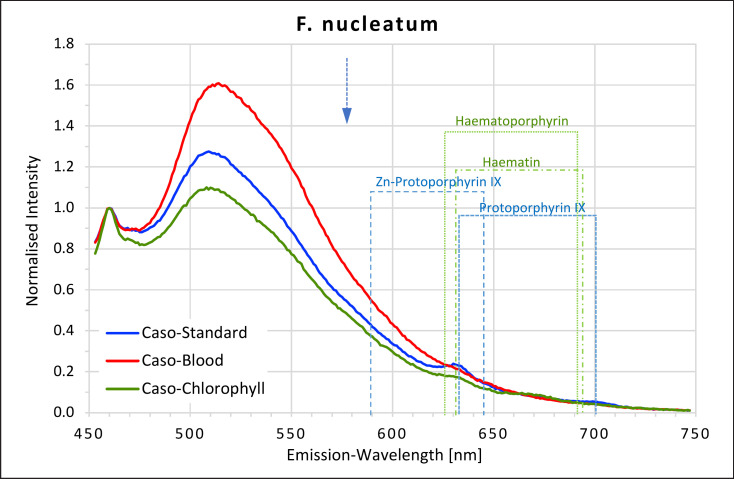
Fluorescence emission spectra for *F. nucleatum* at 405 nm excitation on caso, caso blood, and caso chlorophyll agars. The position of peaks measured for protoporphyrin IX, Zn-protoporphyrin IX, haematoporphyrin, and haematin are shown in dotted lines. Blue arrow at 577 nm.

A large amount of intense green fluorescence with a peak between 505 nm and 510 nm was recorded for this species on all three of the growth media (Fig. 5). The green fluorescence intensity was higher than that measured for the growth media alone and was most pronounced on caso blood agar. No red fluorescence peaks were recorded for this species when grown on caso blood agar. A small peak was found at 630–631 nm, corresponding to the first peaks for protoporphyrin IX and haematin, when grown on caso standard and on caso chlorophyll agars. On caso standard agar, a plateau was found at 700 nm which would correspond to the second peak for protoporphyrin IX.

### L. casei

**Fig. 6:**
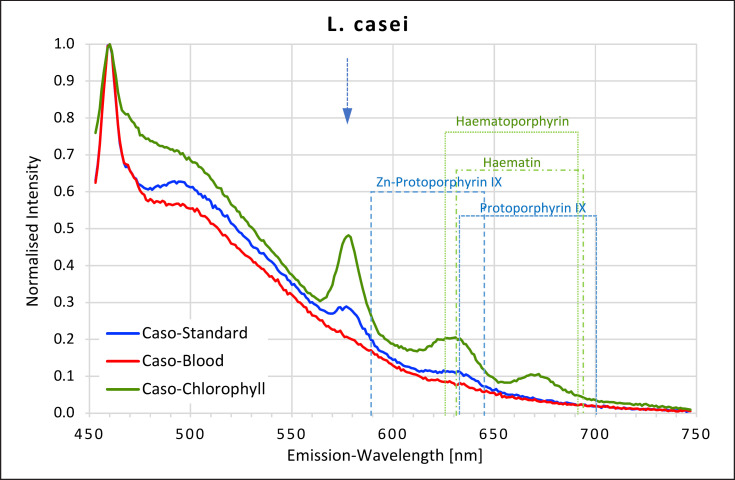
Fluorescence emission spectra for *L. casei* at 405 nm excitation on caso, caso blood, and caso chlorophyll agars. The position of peaks measured for protoporphyrin IX, Zn-protoporphyrin IX, haematoporphyrin, and haematin are shown in dotted lines. Blue arrow at 577 nm.

Three clear peaks were found for *L. casei* colonies when grown on caso chlorophyll agar. Peaks similar to the first two were also found when grown on caso standard agar. But when grown on caso blood agar, only a negligible plateau between 626 and 631 nm was found (Fig. 6).

The peaks around 630 nm match maxima recorded for haematoporphyrin, haematin, and protoporphyrin IX. Neither the first peak at 577 nm nor the third smaller peak between 668 and 672 nm on caso chlorophyll agar could be matched with any of the porphyrins measured. Red fluorescence was inhibited when *L. casei* was grown on blood agar.

### Streptococci

**Fig. 7:**
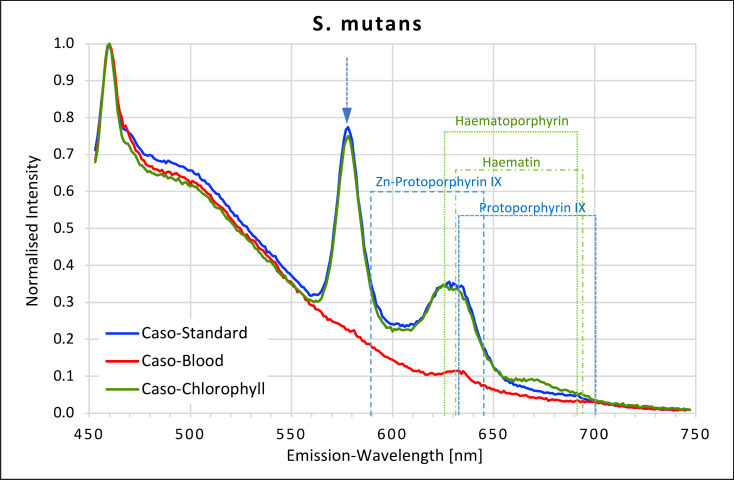
Fluorescence emission spectra for *S. mutans* at 405 nm excitation on caso, caso blood, and caso chlorophyll agars. The position of peaks measured for protoporphyrin IX, Zn-protoporphyrin IX, haematoporphyrin, and haematin are shown in dotted lines. Blue arrow at 577 nm.

**Fig. 8:**
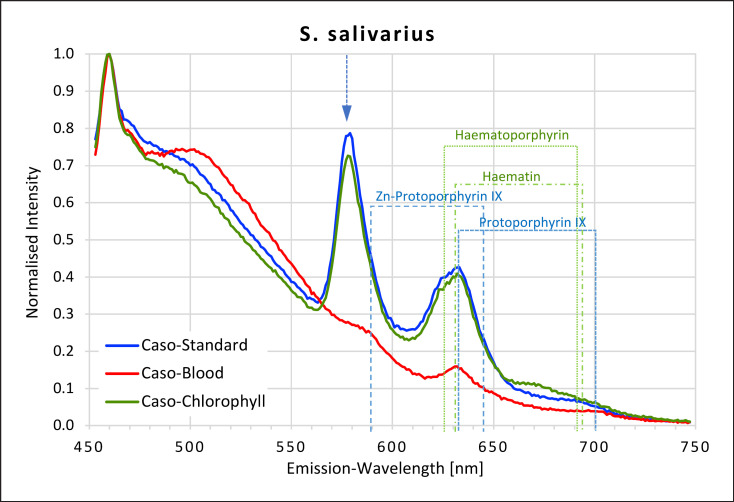
Fluorescence emission spectra for *S. salivarius* at 405 nm excitation on caso, caso blood, and caso chlorophyll agars. The position of peaks measured for protoporphyrin IX, Zn-protoporphyrin IX, haematoporphyrin, and haematin are shown in dotted lines. Blue arrow at 577 nm.

**Fig. 9:**
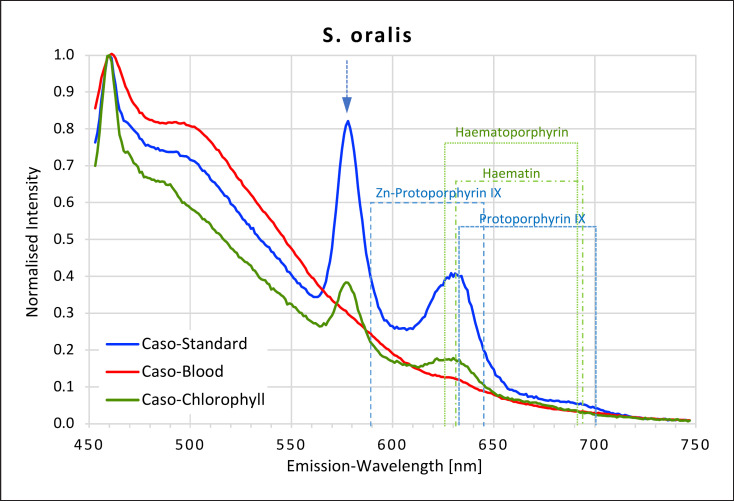
Fluorescence emission spectra for *S. oralis* at 405 nm excitation on caso, caso blood, and caso chlorophyll agars. The position of peaks measured for protoporphyrin IX, Zn-protoporphyrin IX, haematoporphyrin, and haematin are shown in dotted lines. Blue arrow at 577 nm.

**Fig. 10:**
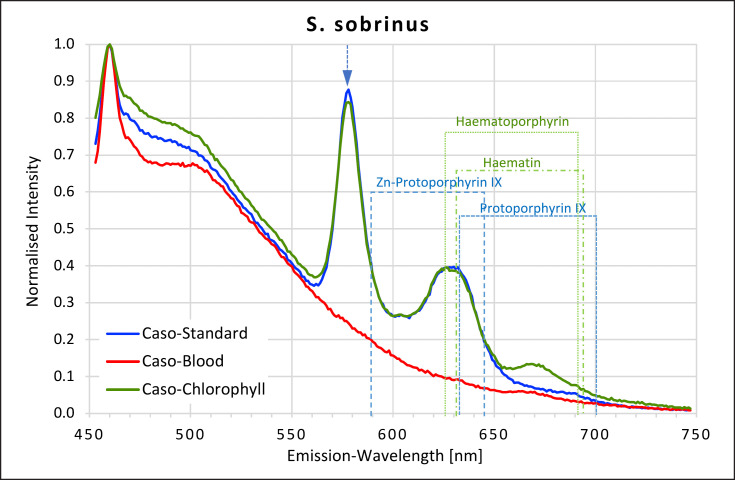
Fluorescence emission spectra for *S. sobrinus* at 405 nm excitation on caso, caso blood, and caso chlorophyll agars. The position of peaks measured for protoporphyrin IX, Zn-protoporphyrin IX, haematoporphyrin, and haematin are shown in dotted lines. Blue arrow at 577 nm.

All *Streptococci* showed high fluorescence intensity peaks in the yellow-red area of the spectrum when grown on caso and caso chlorophyll agars but not when grown on caso blood agar (Fig. 7, 8, 9, 10). The emission spectra of the caso standard agar-grown *Streptococcus* colonies each showed a uniformly identical fluorescence maximum for all 4 strains at 577 nm and a second maximum between 627 and 633 nm matching those for haematoporphyrin, haematin, and protoporphyrin IX. These maxima were also present on caso chlorophyll agar.

In all *Streptococci*, except *S. oralis,* a further small plateau was visible between 666 and 676 nm on caso chlorophyll agar. When grown on caso blood agar, *S. mutans* and *S. salivarius* both exhibited smaller peaks at 631–633 nm, corresponding to haematin and protoporphyrin IX.

### Peak at 577 nm

A clear and very intense peak was measured at 577 nm for all the *Streptococci*, *A. naeslundii*, and *L. casei* but only when grown on caso and caso chlorophyll agars and not on caso blood agar. This peak was not found for either *F. nucleatum* or *A. israelii*. A plateau was found at this wavelength for *P. intermedia* on caso and caso chlorophyll agars. This peak could not be matched with any of the porphyrins tested.

### Plateau at 666–676 nm

A smaller plateau was found between 666 and 676 nm for *S. mutans*, *S. salivarius*, *S. sobrinus*, *A. israelii*, *L. casei*, and *F. nucleatum* but only on caso chlorophyll agar. This plateau could not be attributed to any of the porphyrins included in this study.

## Discussion

This study compared the fluorescence spectra produced by a selection of commonly found oral bacteria associated with both health and disease with those of known porphyrins [Marsh and Martin, 1992]. Species associated with health in one niche may be associated with disease in another. *S. oralis* and *S. salivarius* are part of the normal oral flora but can rarely cause opportunistic infections in other parts of the body. Some cariogenic bacteria like *S. mutans* and *Lactobacilli* are associated with health regarding periodontitis most likely due to their ability to cause a reduction in pH. In the same way, some periodontal pathogens like *F. nucleatum* have been associated with health regarding caries [Zaura et al., 2014].

To date, information on which porphyrins are produced by specific oral microbes is scarce. Protoporphyrin IX is one of the most frequently identified but not the only porphyrin produced by oral bacteria [Buchalla et al., 2008]. Soukos et al. [2005] have shown that the most common porphyrins produced by oral black-pigmented bacteria are protoporphyrin followed by coproporphyrin and small amounts of uroporphyrin. A more recent study found only uroporphyrin and heptacarboxyl porphyrin in *Fusobacterium* cultures [Fontana et al., 2015].

The 405 nm excitation wavelength was chosen because it resembles the Soret band, that is the main excitation band of a huge variety of porphyrin compounds and has been identified as the optimal wavelength for excitation of red caries autofluorescence that is used in numerous caries diagnostic tools [Lennon et al., 2002; Buchalla et al., 2004; de Joselin de Jong et al., 2009; Gomez et al., 2016; Macey et al., 2020]. The range of porphyrins and protoporphyrins included for direct comparison has previously been established as the main source of red fluorescence in carious dentin [Buchalla et al., 2008].

The bacteria were incubated on 3 different growth media. Caso agar was chosen as a base agar because it lacks both *X* and *V* factors (haemin and NAD+/NADP). Peptones contained in caso agar are a source of amino acids, including glycine. Bacteria produce 5-aminolevulinic acid (ALA) from glycine as a first step and can go on to form haem, producing various porphyrins as intermediates via the classical or the non-canonical pathway [Choby and Skaar, 2016]. Caso blood agar, on the other hand, contains 5% sheep's blood, providing haem. Caso chlorophyll agar, prepared from spinach extract, was used to provide another potential porphyrin precursor in the form of chlorophyll, which contains a fifth ring in addition to the tetrapyrrole structure with a magnesium ion at its centre. Spinach also contains vitamins A, B_1-6_, C, E, and K and minerals including iron, copper, zinc, and potassium, providing further sources of both porphyrin ring structures and metals, so chlorophyll is not an exclusive source of porphyrin precursors in this case [Nemzer et al., 2021].

Most previous studies on fluorescence of oral bacteria have used agars containing blood and/or haematin and vitamin K to provide substrates for fluorophore production [Lennon et al., 2006; Zhu et al., 2015]. So far, only one other group has looked at the effect of metalloporphyrins in growth media on fluorescence also using spinach extract to introduce chlorophyll to the nutrient agar [Volgenant et al., 2013]. In contrast to this group, we found that quite some species, notably the *Streptococci*, produced red fluorophores when grown on caso standard agar in the absence of blood or spinach extract.

A clear peak was detected at 577 nm for several species but only when grown on caso and caso chlorophyll agars. This peak in the yellow area of the visible spectrum cannot be attributed to any of the porphyrins we included in the study. The 577 nm peak is most likely caused by a metalloporphyrin. A fluorescent component with emission at around 580 nm has been described by Melø et al. [1985] in the emission spectra for *Propionibacterium acnes* at 415 nm excitation. In a further study, this peak was shown to be separate from those recorded for coproporphyrin III and protoporphyrin IX but thought to be derived from coproporphyrin III because of the reduction of the coproporphyrin peak, with simultaneous increase in the 580 nm peak after storage in darkness. The incorporation of a metal ion, such as copper, zinc, or potassium, which is available in spinach but also in blood, into a porphyrin or coproporphyrin may explain this shift in emission maxima.

In general, we found that the nutritional dependent fluorescence behaviour of the strains could be split into two groups with porphyrin production being inhibited or absent either on blood or on chlorophyll agar. For example, in *Streptococci,L. casei*, and *F. nucleatum* porphyrin production was inhibited on agar containing blood. In *P. intermedia* and *A. naeslundii* porphyrin production was inhibited on agar containing spinach extract.

The *Streptococci* and *L. casei* produced strong fluorescent peaks when grown on caso standard or caso chlorophyll agar plates but not on caso blood agar. This confirms previous research where red fluorescence was absent for *Streptococci* grown on blood agar, although this was recorded as a visual impression rather than spectroscopy [Lennon et al., 2006]. The first peak at 577 nm, most likely indicates an unidentified but specific porphyrin. A shoulder between 627 nm and 633 nm for both *Streptococci* and *L. casei* corresponds with peaks found for haematoporphyrin, haematin, and protoporphyrin IX. *F. nucleatum* produced not only strong green fluorescence as reported earlier but also red fluorophores when grown on caso standard or caso chlorophyll agars [Lennon et al., 2006]

*P. intermedia* and *A. naeslundii* on the other hand produced fluorescence peaks matching Zn protoporphyrin IX, protoporphyrin IX, and haematin when grown on blood or caso agar. This effect was missing on caso chlorophyll agar. The actinomyces behaved differently with *A. naeslundii* producing three different porphyrin profiles on each of the three agars and *A. israelii* producing small peaks matching haematoporphyrin, haematin, and protoporphyrin IX but only on caso chlorophyll agar.

It is now known that the bacterial haem biosynthesis pathways for most gram-positive (via the coproporphyrin III intermediate) and most gram-negative (via the protoporphyrinogen IX intermediate) species differ [Mayfield et al., 2011]. But neither this nor differences in aerobic fastidiousness can explain the inhibition on different nutrient agars as all these combinations were found both in the species with porphyrin inhibition on blood agar and in those with inhibition of fluorescence on chlorophyll agar.

During an infection, the human body sequesters haem so that less is available for invading microbes [Anzaldi and Skaar, 2010]. To combat this, all bacteria have evolved mechanisms for the capture and uptake of haem but also for its excretion to prevent iron toxicity [Choby and Skaar, 2016].

It is possible that it is not the absence of a substrate but the presence of one in excess, for example, haem in blood agar that results in the inhibition of fluorophore production via a feedback mechanism. Since haem is the end product, its overabundance is likely to downregulate its production, explaining the lack of fluorescence in some species when grown on blood agar.

Each of the porphyrins measured in this study produced both first and second peaks when measured individually, but usually only one peak could be identified from the bacterial samples due to overlapping by other peaks. Some porphyrins present may not have been identified for this reason. On the other hand, some peaks were identified in addition to the porphyrins we included, indicating that in future a larger range of porphyrins and metalloporphyrins should be included for this type of investigation. Another limitation is that spectra were measured at only one point in time although porphyrin production is known to shift over time due to pH changes or passaging [Fyrestam and Östman, 2017]. The fact that porphyrin production differs for individual microorganisms opens the possibility of using fluorescence methods to identify specific groups of bacteria in plaque or dentin. Furthermore, haem synthesis and transport pathways are of interest as potential antimicrobial targets in the future.

## Conclusions

Oral bacteria involved in caries and periodontitis showed red fluorescence emission matching known porphyrins, depending on nutrients available in the culture medium. Many oral bacteria synthesise porphyrin compounds without using tetrapyrroles already existing in the agar. Non-red-fluorescing porphyrin-like precursors (haem, chlorophyll) may facilitate or inhibit synthesis of red-fluorescing porphyrin compounds. The cause for inhibition of red fluorescence on blood or chlorophyll agar in some species is currently not known. *F. nucleatum* synthetized primarily green fluorescing compounds under the conditions tested in this study.

## Statement of Ethics

Ethical approval is not required for this study in accordance with local or national guidelines.

## Conflict of Interest Statement

There are no conflicts to declare.

## Funding Sources

There was no funding for this study. The author Áine Lennon received a fellowship from the Alexander von Humboldt Foundation (Germany) between 2003 and 2005, before the beginning of this study. The Alexander von Humboldt Foundation played no role in the preparation of data or the manuscript.

## Author Contributions

Conception and design: Wolfgang Buchalla and Áine Lennon; data acquisition, analysis, and interpretation: Leonora Brune, Simone Techert, Áine Lennon, and Wolfgang Buchalla; drafted the manuscript: Áine Lennon and Wolfgang Buchalla; revised the manuscript: Áine Lennon, Wolfgang Buchalla, Leonora Brune, and Simone Techert.

## Data Availability Statement

All data generated or analysed during this study are included in this article. Further enquiries can be directed to the corresponding author.
